# Development of a *Brassica napus* (Canola) Crop Containing Fish Oil-Like Levels of DHA in the Seed Oil

**DOI:** 10.3389/fpls.2020.00727

**Published:** 2020-06-12

**Authors:** James R. Petrie, Xue-Rong Zhou, Antonio Leonforte, Jason McAllister, Pushkar Shrestha, Yoko Kennedy, Srinivas Belide, Greg Buzza, Nelson Gororo, Wenxiang Gao, Geraldine Lester, Maged P. Mansour, Roger J. Mulder, Qing Liu, Lijun Tian, Claudio Silva, Noel O. I. Cogan, Peter D. Nichols, Allan G. Green, Robert de Feyter, Malcolm D. Devine, Surinder P. Singh

**Affiliations:** ^1^CSIRO Agriculture and Food, Canberra, ACT, Australia; ^2^Nuseed Pty Ltd., Horsham, VIC, Australia; ^3^Nuseed Pty Ltd., Laverton North, VIC, Australia; ^4^Nuseed Americas Inc., Woodland, CA, United States; ^5^CSIRO Agriculture and Food, Werribee, VIC, Australia; ^6^CSIRO Manufacturing, Clayton, VIC, Australia; ^7^Nufarm Ltd., Laverton North, VIC, Australia; ^8^Department of Primary Industries, Melbourne, VIC, Australia; ^9^CSIRO Ocean & Atmosphere, Hobart, TAS, Australia; ^10^Nuseed Global, Calgary, AB, Canada

**Keywords:** ω3 long-chain polyunsaturated fatty acid, DHA, canola, elite event, field trial

## Abstract

Plant seeds have long been promoted as a production platform for novel fatty acids such as the ω3 long-chain (≥ C_20_) polyunsaturated fatty acids eicosapentaenoic acid (EPA) and docosahexaenoic acid (DHA) commonly found in fish oil. In this article we describe the creation of a canola (*Brassica napus*) variety producing fish oil-like levels of DHA in the seed. This was achieved by the introduction of a microalgal/yeast transgenic pathway of seven consecutive enzymatic steps which converted the native substrate oleic acid to α-linolenic acid and, subsequently, to EPA, docosapentaenoic acid (DPA) and DHA. This paper describes construct design and evaluation, plant transformation, event selection, field testing in a wide range of environments, and oil profile stability of the transgenic seed. The stable, high-performing event NS-B50027-4 produced fish oil-like levels of DHA (9–11%) in open field trials of T_3_ to T_7_ generation plants in several locations in Australia and Canada. This study also describes the highest seed DHA levels reported thus far and is one of the first examples of a deregulated genetically modified crop with clear health benefits to the consumer.

## Introduction

The long-chain (≥ C20) polyunsaturated fatty acids (LC-PUFA) such as eicosapentaenoic acid (EPA; 20:5ω3) and docosahexaenoic acid (DHA; 22:6ω3) are essential components of animal cell membranes and important contributors to human health. Certain tissues and organs, including eyes, brain and heart, are particularly rich in EPA and DHA and there is an abundance of evidence supporting the beneficial role of EPA and/or DHA in promoting eye health, heart health and cognitive function (Simopoulos, 1999; Martin, 2011; Hashimoto et al., 2017). Although primary production of these fatty acids occurs predominantly in ocean-based microalgae and some bacteria, the major sources of EPA and DHA for human consumption are oil extracted from various fish species, either wild or farmed (for example, salmon, trout, anchoveta, menhaden, etc.) that accumulate these LC-PUFA through the marine food web or through their supplied diet (Abedi and Sahari, 2014). Various assessments indicate that most humans consume lower than optimal amounts of EPA and DHA required, and the total ocean supply of LC-PUFA is insufficient to meet the recommended daily intake for the global population (Stark et al., 2016). Rising human populations and increased awareness of the health benefits of LC-PUFAs on one hand, and restrictions on the allowable catch of the marine sources of LC-PUFAs on the other, are expected to create a shortage of these key nutrients in the near future (Adarme-Vega et al., 2014; Sprague et al., 2017).

There is a compelling sustainability argument in favor of supplementing the marine supply of LC-PUFA with a land-based source (Napier et al., 2015). High oil yield and relatively low production costs of oil crops would provide economic and sustainable production platform of oil containing EPA and DHA. We identified canola (*Brassica napus* L.) as a potential oil crop for EPA and DHA production. Canola seed production is up to 4 T.ha^–1^, with 40–45% seed oil content. It has broad agronomic and geographic adaptation, great genetic resources and substantially developed germplasms. If canola can be used producing DHA at levels of 10–15% in its oil, 2.5 million ha of such canola (about 2% of global cultivation of major oilseed crops) would provide the equivalent amount of DHA currently harvested from all fish oils (Zhou et al., 2019).

**FIGURE 1:**
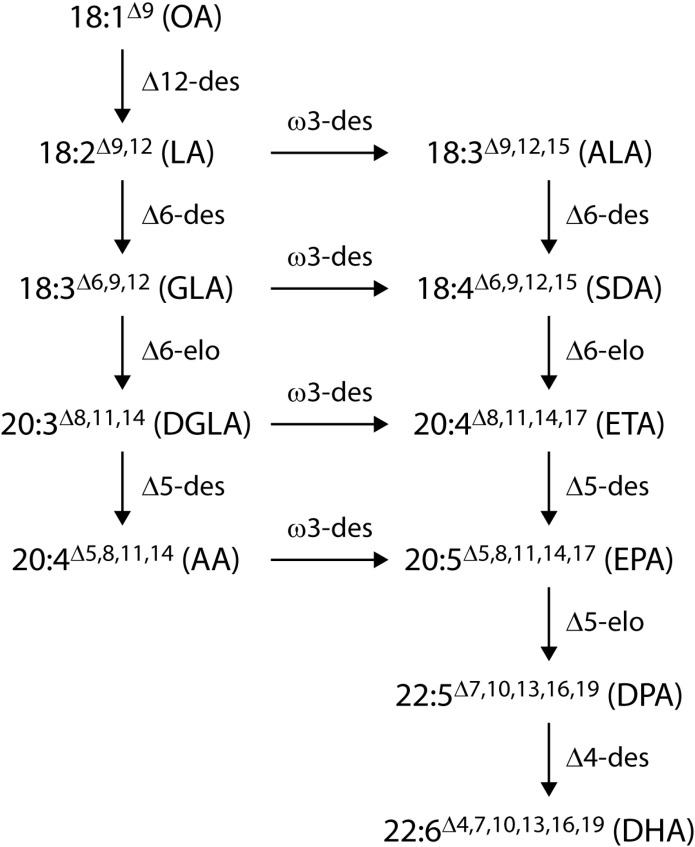
The Δ6-desaturase aerobic long-chain (≥ C20) polyunsaturated fatty acid synthesis pathways. Desaturases are denoted by ‘des’ and elongases by ‘elo’. The ω6 pathway runs in parallel with the ω3 pathway with fatty acids converted across by the action of an ω3-desaturase. Abbreviations are OA, oleic acid; LA, linoleic acid; ALA, α-linolenic acid; GLA, γ-linolenic acid; SDA, stearidonic acid; DGLA, dihomo-γ-linolenic acid; ETA, eicosatetraenoic acid; AA, arachidonic acid; EPA, eicosapentaenoic acid; DPA, docosapentaenoic acid; DHA, docosahexaenoic acid.

In the past 15 years, two approaches have been taken to produce EPA and DHA in oil crops: first, the expression of the microalgal polyketide synthase system (Metz et al., 2001) which synthesizes DHA from malonyl-CoA (Walsh et al., 2016) and, second, the stepwise extension of the endogenous higher plant lipid biosynthetic system through addition of the alternate fatty acid desaturases and elongases from the more conventional aerobic pathway to produce EPA and DHA (Robert et al., 2005; Wu et al., 2005; Petrie et al., 2012; Ruiz-Lopez et al., 2014). Substantially higher total levels of EPA and DHA have been reported in plant seeds by engineering the aerobic desaturase and elongase pathway, particularly the Δ6-desaturase mediated version of this pathway (Figure 1). The successful accumulation of EPA and DHA into the seed oil of plant hosts by this method has been reported in the model species *Arabidopsis thaliana* (Robert et al., 2005; Petrie et al., 2012). This technology was subsequently transferred to *Camelina sativa* as a minor oilseed crop by assembling five to seven genes from marine microalgae and fungi to engineer an EPA-alone or an EPA+DHA oil (Ruiz-Lopez et al., 2014). Independent work by our group, harnessing expression of a different set of seven transgenes from yeast and microalgae, demonstrated DHA production of up to 12.4% of total fatty acids in oil from transgenic *C. sativa*, similar to the level found in DHA-rich fish oil, and an EPA content of 0.8–3.3% (Petrie et al., 2014). More recently, Usher et al. (2017) reported 16–17% EPA, and 4–5% EPA plus 4% DHA in field tests of an EPA and DHA constructs, respectively, in field-grown *C. sativa*.

In this paper we report the successful translation of our high DHA technology, initially demonstrated in *Arabidopsis* and *C. sativa*, into canola (*B. napus*), the world’s second most important annual oilseed crop, to provide a highly scalable platform for commercially sustainable DHA production. We describe construct design and evaluation, plant transformation, event selection, field testing in a wide range of environments, and stability testing of the transgenic seed and oil. The completion of molecular, biochemical, genetic stability and agronomic characterisation of this canola event has resulted in regulatory approval for cultivation of the crop in Australia for human and animal consumption of the oil (FSANZ, 2017; OGTR, 2018) and cultivation approval in the USA. Similar approvals are pending in Canada.

## Materials and Methods

### Oligonucleotides

The oligonucleotides used in this work were chemically synthesized by Sigma-Aldrich (Castle Hill, NSW, Australia). The oligonucleotide sequences are listed in Supplementary Table 1.

### Plasmid Construction

**FIGURE 2:**
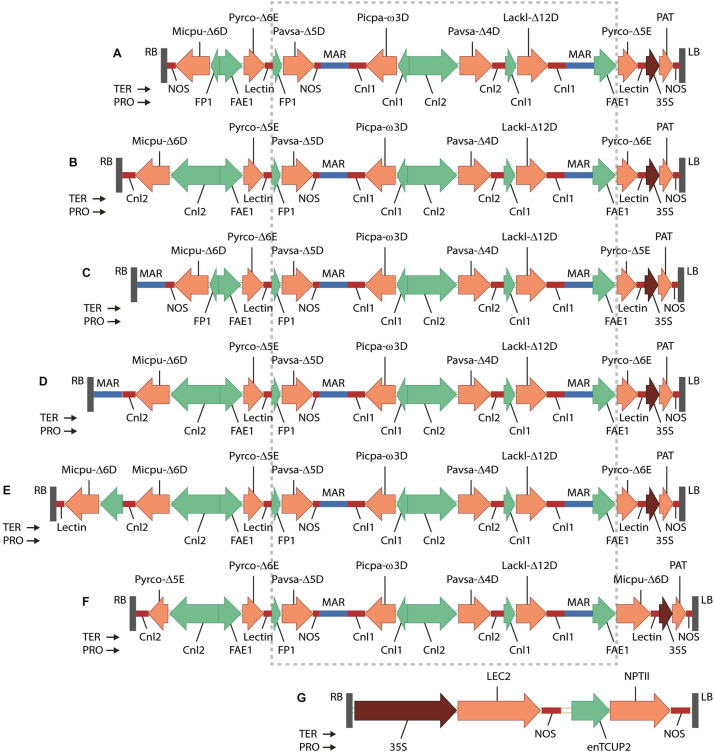
T-DNA regions of binary vectors tested in this study shown in the right border (RB) to left border (LB) orientation: **(A)** GA7; **(B)** GA7_mod-B; **(C)** GA7_mod-D; **(D)** GA7_mod-E; **(E)** GA7_mod-F; **(F)** GA7_mod-G; **(G)** pJP3129. The boxed region of the GA7 series is common to all constructs. Genes are: Micpu-Δ6D, *Micromonas pusilla* Δ6-desaturase; Pyrco-Δ6E, *Pyramimonas cordata* Δ6-elongase; Pavsa-Δ5D, *Pavlova salina* Δ5-desaturase; Picpa-ω3D, *Pichia pastoris* ω3-desaturase; Pavsa-Δ4D, *P. salina* Δ4-desaturase; Lackl-Δ12D, *Lachancea kluyveri* Δ12-desaturase; Pyrco-Δ5E, *P. cordata* Δ5-elongase; PAT, phosphinothricin N-acetyltransferase from *Streptomyces viridochromogenes*. Promoters are: FP1, truncated napin promoter from *Brassica napus*; FAE1, *Arabidopsis thaliana* FATTY ACID ELONGATION1 promoter; Cnl1, *Linum usitatissimum* CONLININ1 promoter; Cnl2, *L. usitatissimum* CONLIIN2 promoter; 35S, Cauliflower mosaic virus 35S promoter with duplicated enhancer region. Terminators are: NOS, *Agrobacterium tumefaciens* NOS; Lectin, *Glycine max* Lectin; Cnl1, *L. usitatissimum* conlinin1; Cnl2, *L. usitatissimum* conlinin2. MAR denotes the Rb7 matrix attachment region from *Nicotiana tabacum*.

A set of binary vector GA7 (Petrie et al., 2012) variants GA7_mod-B, GA7_mod-D, GA7_mod-E, GA7_mod-F and GA7_mod-G were constructed. The seven fatty acid biosynthesis genes were chemically synthesized at GeneArt (Regensburg, Germany) as a single 19,750 bp fragment flanked with *Not*I sites, which was then cloned into vector pJP3416 to generate GA7 (Petrie et al., 2012). The vector GA7_mod-B was built by re-arranging the two elongase genes and replacing the promoter for Micpu-Δ6D in the existing vector GA7 (Figure 2A). A fragment with Pro-FAE1::Pyrco-Δ6E::Ter-Lectin was first cloned into the two *Sbf*I sites of GA7 to replace Pro-FAE1::Pyrco-Δ5E::Ter-Lectin near the left border with Pyrco-Δ6E, generating intermediate GA7_mod-A. The original Micpu-Δ6D expression cassette and the adjacent Pyrco-Δ6E in GA7_mod-A was replaced with a *Pme*I-*Asc*I fragment containing Pro-Cnl2::Micpu-Δ6D::Ter-Cnl2 and Pro-FAE1::Pyrco-Δ5E to yield the final vector, GA7_mod-B (Figure 2B). A fragment containing *Nicotiana tabacum* Rb7 matrix attachment region (MAR) was inserted at *Pme*I site between right border and Micpu-Δ6D expression cassette in GA7, generating GA7_mod-D (Figure 2C). The same MAR fragment was also inserted into same *Pme*I site between right border and NOS terminator in GA7_mod-B, generating GA7_mod-E (Figure 2D). The variants GA7_mod-F and GA7_mod-G were previously reported (Petrie et al., 2014; Figures 2E,F). All these binary vectors were transferred to *Agrobacterium tumefaciens* strain AGL1 (Lazo et al., 1991).

### Expression of Seed Specific Constructs in LEC2-Induced Canola Somatic Embryos

*B. napus* somatic embryos were generated by co-transformation of a 35S::LEC2 vector with a seed-specific GA7 construct variant as previously described (Belide et al., 2013). The DHA levels as a percentage of the somatic embryo total fatty acids were analyzed by gas chromatography (see below). The conversion efficiencies of each enzymatic step involved in DHA biosynthesis pathway are calculated as the sum of the products divided by the sum of the substrate and the products.

### Canola Stable Transformation

Several canola varieties were used as transformation recipients, including the ‘Oscar’ cultivar and elite commercial varieties such as AV-Jade, AV-Spectrum, AV-Zircon, and the high-oleic ‘Monola’ variety. *B. napus* cotyledonary petioles were transformed with the binary vector GA7_mod-B essentially as described by Belide et al. (2013) with slight modification. Briefly, infected cotyledonary petioles were cultured on MS media as described (Belide et al., 2013) except using 5 mg L^–1^ PPT for selection with a biweekly subculture. Shoots from the resistant callus were transferred to shooting media (MS media with 0.1 mg L^–1^ gibberelic acid + 3 mg L^–1^ AgNO_3_ + 250 mg L^–1^ cefotaxime + 5 mg L^–1^ PPT) to extend the shoots for another two weeks. Selected healthy shoots with one or two leaves were transferred to rooting media (1/2 MS with 1 mg L^–1^ NAA + 20 mg L^–1^ ADS + 3 mg/mg L^–1^ AgNO_3_ + 250 mg L^–1^ cefotaxime). DNA was isolated from leaf with plant DNA isolation kit (Bioline, Alexandria, NSW, Australia) to confirm the T-DNA insertion by PCR. The confirmed lines were transferred to pots and grown in glasshouse.

### Characterization of T-DNA Copy Number by Digital PCR

Genomic DNA was isolated from leaf tissues as described by Mieog et al. (2013) using TissueLyser II (Qiagen, Hilden, Germany). Primers and probes were designed with Primer 3 plus^1^ (Rozen and Skaletsky, 1999) and tested in AutoDimer^2^ to study the potential interaction between the reference and target oligonucleotide trio (Supplementary Table 1). High-mobility-group (HMG) protein, a single copy in the genome of *Brassica* species (Weng et al., 2004), was used as a reference. The primer set for HMG protein (HMG-F/HMG-R) combined with the HMG-P probe was designed to detect a 73 bp of HMG. As for the target genes, we used PPT selectable marker and delta-6 desaturase (Δ6D) fragment, positioned at the left and right borders, respectively, in GA7_mod-B. The PPT-F/PPT-R primer pair combined with PPT-P, and the Δ6D-F/Δ6D-R primer pair combined with Δ6D-P, were used to amplify fragments of 82 and 89 bp, respectively. The reference gene probe was labeled with 5′HEX (hexachloro-fluorescein) and the transgene probes were labeled with 5′FAM (6-fluorescein). Probes were doubled-quenched with ZEN^TM^ and Iowa Black Hole Quencher 1. We obtained primers and probe from SIGMA and Integrated DNA Technologies Inc., respectively.

A range of 50 to 100 ng genomic DNA was digested with 4 units each of *Bam*HI and *Eco*RI (New England Biolabs, Ipswich, MA, United States) in a final volume of 20 μL, at 37°C, 4 h to overnight. Digested DNA was added to the Bio-Rad 2XddPCR^TM^ Supermix for Probes (no dUTP) (Bio-Rad, Gladesville, Australia) at concentrations ranging from 20 to 120 ng DNA per 20 μL dd-PCR reaction. TaqMan assay primers and probes were used at the final concentrations of 900 and 250 nM, respectively. For triplex assay, Δ6D-P was reduced to 50 nM. Samples were placed onto Droplet Generator QX200^TM^ (Bio-Rad) or QX200AutoDG and heat sealed with a pierceable foil heat seal with PX1 PCR plate sealer (Bio-Rad). Plates were placed in C1000 Thermal Cycler (Bio-Rad) and reactions were run with the following cycles: 95°C for 10 min followed by 40 cycles at 94°C for 30 s; 61°C for 1 min, then 98°C for 10 min and maintained at 12°C finally. The ramping rate of 2.5°C/s in all temperature change steps were used. After amplification, the plates were loaded onto the QX200 Droplet Reader (Bio-Rad). Data analysis was performed using Quanta soft^TM^ software (Bio-Rad).

### Sequencing of T-DNA Integration Site in Elite Events

Genomic DNA of eight T_3_ and T_4_ lines were randomly sheared using the Covaris Technology (Covaris, Woburn, MA, United States) and fragments with size of 700–900 bp were collected. Both ends of the sheared DNA fragments were ligated with a single Illumina adaptor (universal primer PE2, Supplementary Table 1). A total of 78 vector-specific primers (data not shown) were designed to span the whole GA7_mod-B vector (31,564 bp). These primers were ligated to another Illumina adaptor (universal primer PE1, Supplementary Table 1). T-DNA containing fragments were enriched by nested PCRs with the Illumina universal primer PE2 and vector-specific primers, followed by further enrichment through PCR amplification with Illumina universal primer PE2 and the T-DNA linked Illumina universal primer PE1. Sequencing of fragments from both ends (paired end) was performed on an Illumina MiSeq (Illumina, San Diego, CA, United States) with Illumina universal primers PE2 and PE1, generating 2 × 250 bp sequencing reads. More than 250,000 raw reads were generated. The raw reads were trimmed and cleaned filtered with the following criteria by a custom computational script written in Perl. The reads were trimmed if > 3 “N” nucleotides were called, > 3 nucleotides had PHRED quality score ≤ 20, or the median PHRED score ≤ 20. The reads were discarded when sequence read length was < 75 nucleotides, or mate pair was discarded, or if Illumina adaptor was missing. The reads passed the criteria and kept by Perl were then passed through the Cutadapt v1.4.1 (Martin, 2011) program to trim any remaining adaptor sequences. After the two-step quality filtering successfully removed all of these adaptors, about 10% of the raw sequence reads were removed. The clean sequences of the enriched fragments were then aligned to 31,564 bp reference sequences of vector, using BWA software package^3^ and the MEM Algorithm was used to generate T-DNA sequence in FASTA format. The alignments were then converted into an indexed BAM file using the SAMtools software package^4^ and can be visualized in software packages such as Tablet^5^.

The sequence reads aligned to the vector reference at only a single read of the paired-end reads were used for further analysis. Specific sub-sets of reads generated in the right and left border regions were specifically targeted for junction sequence from canola genome by aligning to the reference genome sequences of *B. napus* (2*n* = 4*x* = AACC^6^), *B. rapa* (2*n* = 2*x* = AA^7^) and *B. oleracea* (2*n* = 2*x* = CC^8^). The identified sequence reads were assembled using the CAP3 software^9^, and BLASTed to the reference genomes of *Brassica*. The contigs that had significant matches to the reference sequence of both *Brassica* and the binary vector were used to define the copy number and integration sites of T-DNA inserts in DHA canola.

### Genetic Stability of the T-DNA Inserts in DHA Canola

A total of 100 seeds from T_3_ to T_7_ generation (20 seeds/generation) of DHA canola were used for DNA extraction based on LGC Oktopure DNA Extraction System (LGC, Boston, MA, United States). DNA concentration was measured with NanoDrop 8000 UV-Vis Spectrophotometers (Thermo Fisher Scientific, Wilmington, DE, United States). One non-GMO control (AV-Jade) and one event positive control (transgenic GA7_mod-B *Brassica napus* event) were included. This positive control was previously used to characterize DHA canola through PCR amplicon sequencing.

Four assays (EAD02DJ517, EA05DJ380, EA02UJ284 and EA05UJ200) were designed with Primer3Plus^10^ for primer melting temperature (Tm) of 59–60°C. Each assay was targeting one of the four junction regions of the two T-DNA copies in DHA canola. To facilitate the electrophoresis with four assays simultaneously, the assays were designed with distributed amplicon size (517, 380, 284 and 200 bp). All four assays spanned the junctions of *B. napus* genomic sequence and the T-DNA inserts.

PCR amplification was performed in 25 μL reaction according to the recipe of OneTaq PCR Amplification Kit (New England Biolabs, Ipswich, MA, United States). Touchdown PCR was used for amplification with the following parameters: 1 cycle of 94°C for 30 s, followed by 6 cycles of 94°C for 30 s, 64–58°C (dropping 1.0°C per cycle) for 20 s and 68°C for 30 s, and followed by 33 cycles of 94°C for 30 s, 58°C for 20 s and 68°C for 30 s with an final extension at 68°C for 5 min.

Each DNA sample was PCR amplified individually with each assay. After PCR amplification, 2.25 μL of PCR amplicons from each of the four assays were mixed together with 1.0 μL of 10 × Ambion gel loading buffer (Thermo Fisher Scientific, Wilmington, DE, United States) for electrophoresis on 1.5% agarose gel.

### Field Evaluation

Event NS-B50027-4 was grown in the field at several Australian sites in 2015 and 2016 and in Canada in 2016 to determine both agronomic performance and whether growing conditions affected DHA synthesis and accumulation. AV Jade and other non-transgenic varieties, and the A02 and A05 segregants were also included in the 2016 trials. Canola was grown in open field plots or in pure seed production tests under the relevant isolation and monitoring regulations, and agronomic parameters, including grain and oil yield and the fatty acid profiles of harvested seed were evaluated. The experimental sites were at different locations with varying environments for soil type, growing season rainfall and agronomic management practices. When available, irrigation was supplied on a limited basis at some sites as required to supplement rainfall. In all cases plot size was 10 m^2^ and seeding rate adjusted on the basis of seed size and germination percentage to approximately 35 plants per m^2^ in Australia and 70 plants per m^2^ in Canada. Each trial experiment was designed with a randomized block consisting of 5 replicates with 12–14 entries (combination of transgenic lines and 6–8 check cultivars). The Australian cultivar entries represented a range of agronomic diversity including plant habit, phenology and yield potential in the Australian cropping zones. These cultivars are open pollinated and have been described in the Australian National Variety Testing Program and Regional annual crop reports^11^. AV-Jade is the non-transformed isoline of NS-B0050-24. The two Canadian commercial cultivars (DK7444 and LL130) were high-yielding, herbicide-tolerant hybrids. The transgenic lines included T_3_, T_5_ and T_7_ generations of NS-B0050-24. In 2016, segregant lines containing the A05 or A02 loci were included in the trials.

Agronomic data were collected for each plot in all trials including plant emergence (number of emerged plants per m^–2^ approximately 14 to 24 days post-sowing; plant vigor (estimate of ground cover at about the 6-leaf stage, using a 1 to 9 score, where 1, 5 and 9 represent ≤ 10%, 40–50% and >90% ground cover, respectively); flowering time (number of days from sowing to when 50% of plants had at least one open flower); flowering end time (days from sowing to when 90–95% of plants had no open flowers); flowering duration (difference in days between flowering time and end flowering time); plant height (cm) at maturity; seed shattering (seed collected over a 2-week period in two trays (total 0.12 m^–2^) placed between rows); lodging resistance (1 to 9 score based on plant angle from the base of the plant, where 1 = erect plants, 5 = moderate lodging and 9 = complete lodging); plant stand at harvest (plants in two 1-m^–2^ quadrants within each plot); and plant survival (plant count as a % of site mean for plant emergence).

Severity symptoms of Blackleg leaf from representative pathogens *Leptosphaeria maculans* and *L. biglobosa* were recorded as scores of 1 to 9, where 1 = 0–5%, 5 = 20–25% and 9 ≥ 40% leaf infection, respectively across five sites. Symptoms associated with cankering and stem breakage were not observed. Opportunistic disease or insect stress that could be recorded were also observed in all field trials.

Grain was harvested when seed was physiologically mature and dry (∼7% moisture content) using a plot harvester and yield recorded (metric tons per ha). Grain moisture at harvest was determined on a bulk sample using a hand-held moisture meter (Wile 65 Moisture Meter, Farmcomp Agroelectronics; Australia) or with a Harvest Master Classic Grain Gauge with an Allegro CX (Juniper Systems; Logan, UT, United States).

Restricted estimated likelihood analysis was undertaken on factors measured using ASReml procedures in GenStat (Version 17). A statistical method with linear mixed model was used to account for field spatial variation. This method has been extensively described and used for plant breeding in field and genetics research (Smith et al., 2001).

### Generation of A02 and A05 Segregants

A backcrossing program was undertaken in glasshouses at Nuseed (Horsham, VIC, Australia) to transfer the GMO inserts from NS-B50027-4 to non-GMO canola genotypes. Segregating progeny from the BC1F3 generation varying for the presence or absence of locus inserts (A02, A05) and zygosity were identified using linked molecular markers. The segregant populations were used to investigate the individual and additive contributions of the two loci to the phenotypic expression of higher DHA in mature seed.

### Seed Oil Content Analysis

Oil content was determined using an Spinlock SLK-200 Benchtop NMR Spectrometer (Cordoba, Argentina). Approximately 5–8 g of seed was accurately weighed into an NMR tube for analysis, and seed oil content generated by a software calibration created using 20 reference canola samples with known % oil content. The % oil contents of these 20 samples were determined by gravimetric oil extraction (extraction method based on AOF method AOF 4-1.25).

### Seed Moisture Content

For moisture correction of % oil results (field trial seed), moisture content was determined using an oven drying method based on AOF method 4-1.5. Five grams of pre-weighed seeds were dried in an open tin at 130°C for 1 hr. Samples were cooled in a desiccator for 40 min then weighed and % moisture determined as % loss of mass.

### Fatty Acid Profile Analysis

Fatty acid profile analysis of LEC2-induced somatic embryo and canola seeds harvested from CSIRO glasshouses was performed. For pooled seed, 100 mg of seed was crushed in a mixture of 0.9 mL chloroform/methanol (2/1, v/v) in the argon-filled Safe-lock tubes (2.0 mL; Eppendorf) using a metal ball (0.5 cm diameter) and Reicht Tissue lyser (Qiagen) at a frequency of 22 Hz. The homogenate was transferred to a 2 mL glass vial and the solvents were evaporated under the nitrogen flow on a 40°C hot plate. Fatty acid methyl esters (FAME) of the seed homogenate was carried out by incubating the sample in 0.7 mL of 1 N methanolic-HCl (Supelco) at 80°C for 2 h. After cooling the sample to room temperature, 0.3 mL 0.9% NaCl and 0.6 mL hexane were added and vortexed for 5 min. The FAME layer was separated by centrifuging the mixture at 1700 × *g* for 5 min and transferred to a GC vial. FAME were analyzed by GC as described in Zhou et al. (2011). For single seed analysis, the seed was transferred to the argon-filled 2 mL GC vial and the seed was pressed by a metal rod to crack the seed coat. FAME preparation and analysis were carried out essentially as mentioned above, except the seed was incubated in 0.5 mL 1 N methanolic-HCl and FAME were extracted in 0.3 mL hexane. FAME were transferred to a glass insert inside a GC vial. The somatic embryo was freeze dried. The FAME GC analysis was performed as above.

Fatty acid profile analysis of the field trial harvested seed was performed at Nuseed’s facility in Laverton, VIC, Australia. Pooled seed samples from individual plants (typically 20 seeds) were hand-crushed in a culture tube using a metal crushing tool. Seed samples were ground in a Cole-Parmer 4301-02 water-cooled analytical mill, fitted with a reduction lid for small samples, and a chilled water-circulator attached. Approx. 5–7 g of seed was ground, and a subsample transferred to a culture tube. The seed oil was extracted with petroleum ether and the solvent was then evaporated under a stream of nitrogen. An aliquot of the seed oil was derivatised using thermally assisted hydrolysis and methylation (THM). The derivatising reagent was a 1 in 4 dilution of Meth Prep II (Grace^TM^ Alltech^TM^) with the resulting reagent being 1.25% 3-(trifluoromethyl)phenyltrimethylammonium hydroxide in methanol. In brief, 10 μL of seed oil was transferred to a test tube and 1990 μL of 50:50 chloroform:methanol (with 0.01% BHT) was added and vortexed. 100 μL of the solution was transferred to a 9 mm-mouth GC vial with 400 μL flat-bottomed glass insert. A further 50 μL of 50:50 chloroform:methanol was added followed by 50 μL of 1.25% Meth Prep II solution. The vial was capped, vortexed and placed in a heating block at 40°C for 30 min. The FAME were analyzed using a Shimadzu GC-2010plus fitted with an SGE BPX-70 capillary column (0.32 mm i.d. and 0.25 μm film thickness), a FID, a split/splitless injector and an AOC-20S auto sampler and AOC-20I injector. Hydrogen was the carrier gas and was set to constant linear velocity mode of either 40 or 65 cm/sec. Injection volume was either 0.5 or 1 μL with a split ratio of 25:1. The injector was held at 250°C and the detector was held at 300°C. For standard screening, a 30 m column was used. After injection of 0.5 μL sample, the oven temperature was held at 140°C for 1.2 min, raised to 172°C at a rate of 6°C/min, then raised to 200°C at a rate of 10°C/min, held for 1.8 min and finally raised to 250°C at a rate of 50°C/min and held for 0.5 min. The carrier gas linear velocity was 65 cm/sec. The standard screen provided data for all fatty acids in the long-chain omega 3 synthesis pathway leading from oleic acid to DHA. For detailed profiles, a 60 or 90 m BPX-70 column was used. The 90 m column consisted of a 30 m and a 60 m BPX-70 column joined together with a GlasSeal^TM^ capillary column connector. After injection of a 1 μL sample, the oven temperature was held at 50°C for 4 min, raised to 140°C at 50°C/min, raised to 232°C at 3°C/min and finally raised to 250°C at 50°C/min and held for 1 min. The carrier gas linear velocity was 40 cm/sec. The detailed profile provided fatty acid results for all the major and many of the minor fatty acids. Fatty acid profiles were quantified using area normalization with Shimadzu LabSolutions software.

### Seed Oil Profile Stability

Wild type (cultivar AV-Jade) and transgenic seeds, either freshly harvested or stored at 4°C, 24°C or 32°C for 6 months, were used in the determination of oil quantity and fatty acid profile. Total lipids were extracted from the seeds and the amount and the compostion of fatty acids were quantified by GC analysis.

### Fatty Acid Positional Analysis in Seed Oil and Triacylglycerols

Positional analysis of DHA in purified TAG from T_4_ homozygous seeds was performed by ^13^C NMR analysis as described in Petrie et al. (2014).

## Results

### Metabolic Engineering of *B. napus* to Produce DHA

We designed several candidate constructs (Figure 2) to optimize promoter choice for individual genes as well as the order and orientation of genes in the expression vector. Earlier work had demonstrated the functionality of our selected gene set (*Lachancea kluyveri* Δ12-desaturase, *Pichia pastoris* ω3-desaturase, *Micromonas pusilla* Δ6-desaturase, *Pyramimonas cordata* Δ6-elongase, *Pavlova salina* Δ5-desaturase, *P. cordata* Δ5-elongase, and *P. salina* Δ4-desaturase) in plant seeds (Petrie et al., 2013, 2014). The additional constructs were designed in attempts to optimize both the conversion of native plant fatty acid substrates to DHA and reduce accumulation of intermediate fatty acids. GA7_mod-B (Figure 2B) was produced from the parental construct GA7 (Petrie et al., 2013) using a combination of DNA syntheses to produce new DNA fragments and restriction enzyme cloning to swap the position of the Δ5- and Δ6-elongases. The Δ6-desaturase promoter was also changed from the truncated *B. napus* napin (FP1) promoter to the *Linum usitatissimum* conlinin2 promoter (Truksa et al., 2003). These changes sought to increase the Δ6-desaturation of ALA to SDA and the Δ6-elongation of SDA to ETA; the Δ5-elongase efficiency in the original GA7 construct was exceptionally high, but the relatively low Δ6-elongase activity had resulted in the accumulation of SDA in both *A. thaliana* and *C. sativa*. Additional constructs, GA7_mod-D and GA7_mod-E, were generated from GA7 and GA7_mod-B, respectively, and contained a third matrix attachment region (MAR; Halweg et al., 2005) spacer between the T-DNA right border and the Δ6-desaturase expression cassette. This MAR insertion was designed to ‘insulate’ the Δ6-desaturase from the *B. napus* genomic DNA because the close proximity of transgenes to genomic DNA regulatory elements might impair transgene expression. GA7_mod-G switched the positions of the Δ6-desaturase and Δ6-elongase coding regions in the GA7_mod-B format. Finally, GA7_mod-F was based on GA7_mod-B, but contained an additional Δ6-desaturase expression cassette (*A. thaliana FAE1* promoter::*M. pusilla* Δ6-desaturase::*Glycine max* lectin terminator). This variation was designed to both increase the Δ6-desaturase transcript abundance and broaden the expression window by overlapping the FAE1 promoter (Rossak et al., 2001) with the Cnl2 seed storage protein promoter. Finally, a different codon usage was used for the additional Δ6-desturase coding region to avoid any potential secondary structure and silencing issues. The same plant selectable marker, *Streptomyces viridochromogenes* phosphinothricin-N-acetyltransferase (PAT), was used in all vectors.

*Brassica napus* seedling explants were co-transformed with a 35S::LEC2 binary vector (pJP3129, Figure 2G) and one of the binary vectors GA7_mod-B, GA7_mod-D, GA7_mod-E, GA7_mod-F, or GA7_mod-G. Multiple somatic embryos were generated and their fatty acid profiles analyzed. Representative conversion efficiencies for each construct variant in this somatic embryo assay are shown in Supplementary Table 2. GA7_mod-B embryos were generally found to have a high conversion efficiency from SDA through to DHA, although Δ6-desaturation was consistently low, with only a quarter of the ALA being converted. Δ6-elongation was high in this variant (91.6%) without a concomitant reduction in Δ5-elongation. In contrast, the Δ6-elongase efficiency in GA7_mod-D, which differed from the original GA7 parent construct only in the addition of a MAR spacer at the right border, was lower at 69.8%. Δ6-desaturation was not greatly increased in this variant, although it is difficult to conclude that border spacing is not effective in the somatic embryo assay. The addition of a second Δ6-desaturase in GA7_mod-F did increase activity at this step, indicating that expression level was contributing to conversion levels. Finally, the Δ6-desaturation in GA7_mod-G was reduced as a result of switching the positions of the Δ6-elongase and Δ6-desaturase coding regions. The binary vector GA7_mod-B was selected for large-scale *B. napus* transformation.

### Canola Transformation and Event Selection

**FIGURE 3:**
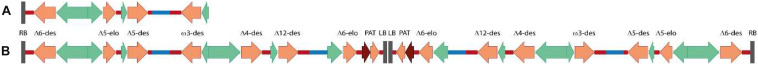
Structure of transgenic loci in *Brassica napus* line NS-B50027-4 following transformation with binary vector GA7_mod-B (Figure 2B): **(A)** Partial T-DNA insertion in chromosome A02; **(B)** Two complete T-DNA inserts in chromosome A05 arranged as an inverted repeat at the left border (linking DNA is shown for illustrative purposes).

Copy number varied greatly across the 1,550 T_0_ *B. napus* lines generated from all varieties, ranging from 0 (presumably selection escapes) to 23, with DHA levels in T_1_ seeds (seeds from the primary transformants) ranging from 0 to 8.7% based on 5 single seeds per line. Supplementary Figures 1A,B show the T-DNA insertion copy number and the DHA levels in T_1_ seeds of 750 representative transgenic T_0_ lines in AG-Spectrum, AV-Jade and Oscar varieties. We selected low-copy (1-2) events useful for backcrossing and breeding. DHA levels in individual transgenic T_1_ seed ranged from 0 to 5.6% of total fatty acids in low-copy events, with both range and average values differing between different canola varieties (Supplementary Figure 1C). High-DHA, low insert copy number T_1_ plants were grown and selfed, and T_2_ seed subjected to a further round of selection on the same criteria. This was repeated for T_2_ plants, using more stringent selection criteria. Genomic DNA from the progeny of several candidate events was isolated and sequenced by Illumina sequencing to determine the structures of the transgenic loci. Ultimately, a single transgenic event, designated by the OECD Identifier NS-B50027-4, was selected for advancement. Transgenic DNA from binary vector GA7_mod-B was inserted in chromosomes A02 and A05 of the canola genome in this event. The transgenic locus at A02 consisted of a partial insertion which included the T-DNA right-border, along with intact Δ6-desaturase, Δ5-elongase, Δ5-desaturase and ω3-desaturase expression cassettes (Figure 3A), while the transgenic locus at A05 was comprised of two complete T-DNA right-border to left-border insertions oriented in a head-head manner (Figure 3B). No vector backbone (vector DNA from beyond the T-DNA border regions) was detected in the genomic DNA of event NS-B50027-4 either by Southern blot (data not shown) or Illumina sequencing of the genome using primers which spanned the complete GA7_mod-B vector. The sequences of the transgene insert and *B. napus* flanking regions at A02 and A05 were deposited in GenBank under accession numbers MP172039 and MP172040.

### Fatty Acid Composition of Transgenic Lines

**FIGURE 4:**
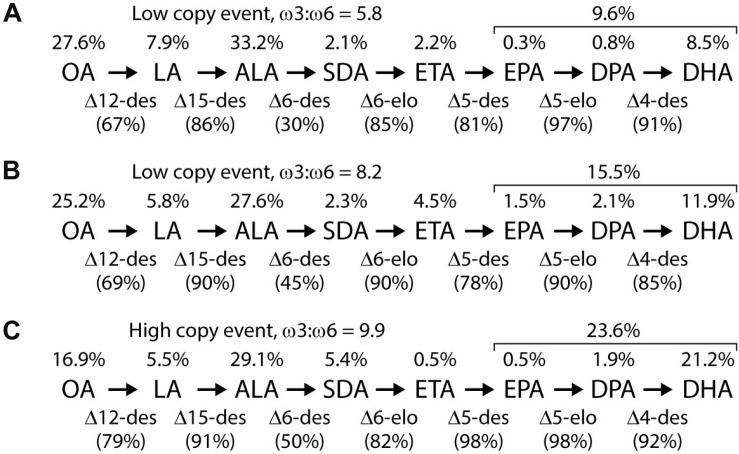
Representative fatty acid profiles of transgenic GA7_mod-B *Brassica napus* events ranging from moderate to high levels of DHA. The bottleneck step is typically Δ6-desaturation, with other enzymatic steps tending to occur at high or very high efficiency. Bracketed values above the pathway describe the total EPA, DPA and DHA levels. Enzyme conversion efficiencies are shown in parentheses and the ω3 to ω6 ratio (all chain lengths) for each event is reported. **(A,B)** Representative events with low copy (1–3 copies), and **(C)** representative event with high copy (> 3 copies) of the T-DNA inserts.

Initial transgenic events contained a wide range in the levels of DHA measured in the seed oil, with levels of 20–25% not uncommon and one event with 34% DHA. However, these tended to be multi-copy events. Multiple lines accumulated DHA in the 10–15% range when homozygous, including progeny from event NS-B50027-4; in comparison, bulk Peruvian anchovy oil, the major marine source, typically contains 11–12% DHA. Omega-3 fatty acid profiles, transgenic enzyme conversion efficiencies, and ω3: ω6 ratios from a range of homozygous mature seed varying in T-DNA copy number are shown in Figure 4, and detailed fatty acid profiles of field-grown NS-B50027-4, the A02 and A05 segregants and the parent, AV-Jade, are shown in Table 1. In general, fatty acid profiles were very consistent across glasshouse and multi-location field trials. As expected, DHA was highest in NS-B50027-4 and found at intermediate levels in plants that contained only the A05 insert. Plants that contained only the A02 partial insert had only trace levels of DHA. No DHA was detected in AV-Jade. Additionally, oleic acid (18:1ω9) content was significantly lower and ALA significantly higher in the transgenic lines. Production of novel ω6 fatty acids was typically very low in the transgenic lines. Total ω3 fatty acids increased in NS-B50027-4 and total ω6 declined, resulting in a considerably higher ω3:ω6 ratio than in the parental line (4-5 cf. < 1). Saturated fatty acid levels remained similar in all lines, at approximately 7–8% of total fatty acids. Of note, no erucic acid (22:1ω9) was produced in NS-B50027-4.

**TABLE 1:** Mean fatty acid profile of field-derived seed oil from transgenic line NS-B50027-4, segregant progeny containing each of the transgenic loci on chromosome A05 or A02, and the parental line AV-Jade.

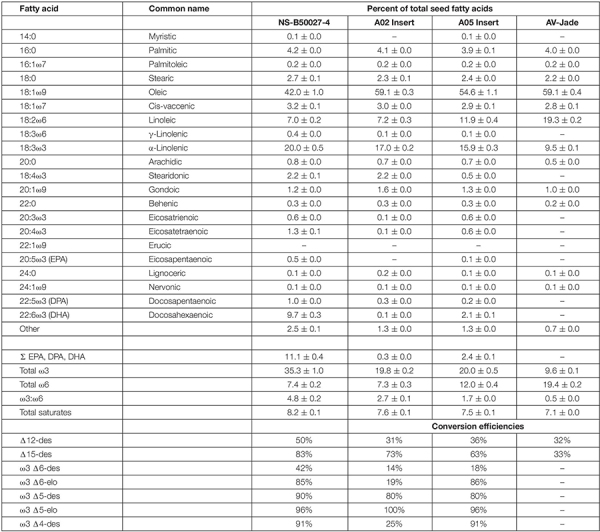

*Data from Ararat, VIC, Australia, 2017; *n* = 5. Conversion efficiencies are calculated as *sum(products)/sum(substrate + products)*. Fatty acids are given as a percentage of seed total fatty acids.*

Interestingly, the lipid class distribution in seed of line NS-B50027-4 was almost identical to that in the parental cultivar Jade: triacylglycerol (TAG) made up 38.9% of total seed mass in NS-B0027-4 (39.6% in Jade), diacylglycerol (DAG) contributed 0.4% (0.3% in Jade), free fatty acids (FFA) contributed 0.3% (0.1% in Jade), and phosphatidylcholine (PC) contributed 1.1% (1.2% in Jade). Retention of DHA and intermediate fatty acids in polar lipids tended to be low relative to retention in the neutral lipids TAG and DAG. DHA was very strongly enriched (97%) in the *sn*-1/3 (α) positions of the TAG molecule (Supplementary Figure 2).

### Field Evaluation

Genotype and environmental conditions (rainfall/available moisture in particular) were the most important determinants of crop yield, resulting in considerable variation in canola yields across sites and years among different varieties tested (Supplementary Table 3). Grain yield of AV Jade was higher than that of NS-B50027-4 in some instances, but not all, indicating that the presence of the transgenic construct did not significantly reduce overall crop yield. There was no significant variation in other agronomic parameters (crop emergence, time to flowering and maturity, pod shattering, disease incidence, pest predation) between NS-B50027-4 and other genotypes included in the field tests. In addition, the junction specific PCR test for the two T-DNA copies in DHA canola from the T_3_ to T_7_ generations indicated that the T-DNA inserts in this event were stably inherited (Supplementary Figure 3).

**FIGURE 5:**
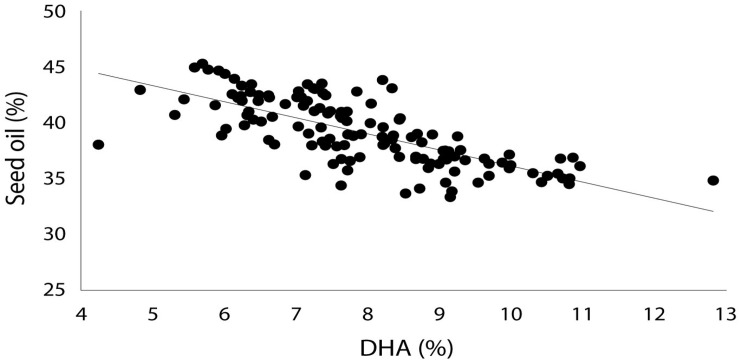
DHA composition (as percentage of total fatty acids in oil) vs. seed oil content (percentage of seed weight) in field-grown GA7_mod-B transgenic *Brassica napus* showing a negative correlation between seed oil yield and DHA composition.

Production of EPA, DPA and DHA in NS-B50027-4 was remarkably consistent across all experimental sites, averaging 0.5, 1.0 and 10% of total seed fatty acid content, respectively. EPA, DPA, DHA, and total LC-PUFA levels from the T_3_-T_7_ generations of NS-B50027-4 are shown in Table 2. T_7_ NS-B50027-4 progeny accumulated 12.5% DHA in open field conditions at Nurrabiel (Victoria, Australia) in 2016. Seed oil content (Supplementary Table 3) was negatively correlated with DHA accumulation (Figure 5) over the range of oil content observed across locations and was reduced by approximately 4% (46% to 42%) in NS-B50027-4. This effect was confirmed by comparing the oil content of NS-B50027-4 with lines that contained only the A02 or A05 locus; NS-B50027-4 (highest DHA) tended to have the lowest oil content, followed by A05 events (intermediate DHA level) and then events only carrying the partial insert in chromosome A02 (no DHA) (Supplementary Table 3).

**TABLE 2:** EPA, DPA, and DHA composition (as percentage of seed total fatty acids) in line NS-B50027-4 over multiple generations.

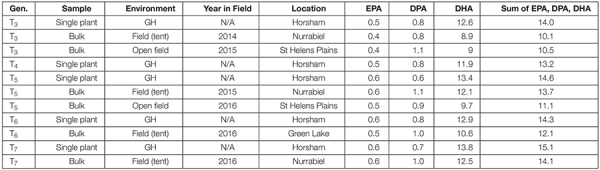

*‘Gen.’ denotes the seed generation, ‘GH’ denotes glasshouse-produced seed; ‘Field (tent)’ denotes pure seed produced in an isolation tent in the field. All sites are in VIC, Australia and were grown in the Australian winter/spring season (May-November/December). Each row shows one measurement from pooled seeds of bulk or single plant at multiple sites with multiple generations.*

**FIGURE 6:**
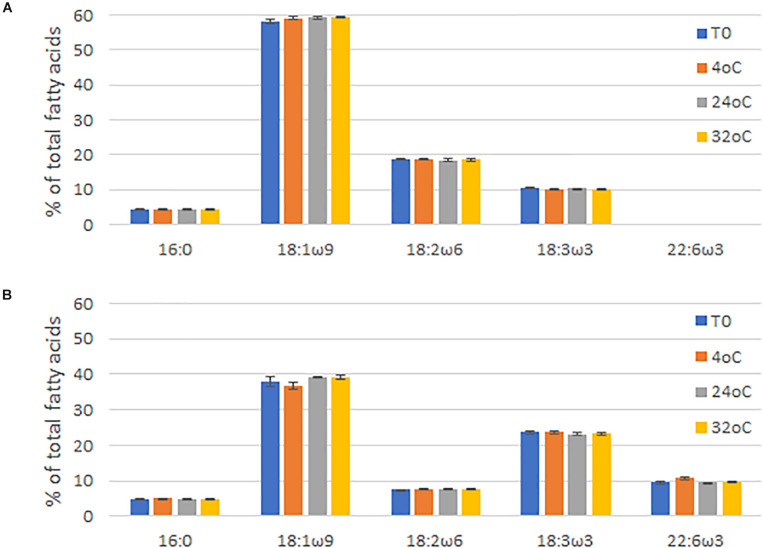
Fatty acid profiles of **(A)** *Brassica napus* AV-Jade cultivar (parental), and **(B)** GA7_mod-B transgenic *B. napus* seeds after being stored for six months at 4°C, 24°C, or 32°C. T0 = time zero (no storage). Data were analyzed by One Way Analysis of Variance (ANOVA) using Sigmaplot (v13) by the Holm-Sidak method. Differences were considered significant at the 5% level. Data are shown as the mean of three repeats ± standard deviation (SD).

Finally, the long-term stability of the seed oil was tested by storing seed from line NS-B50027-4 and Jade at 4°C, 24°C, and 32°C for 6 months and subsequently examining the fatty acid profile of the seed oil. Fatty acid profiles and DHA levels in the seed oil were not significantly affected by storage under these conditions, analyzed by One Way Analysis of Variance (ANOVA), confirming the long-term stability of LC-PUFAs in the canola seed (Figure 6).

## Discussion

We were primarily focused on achieving a high level of DHA accumulation with low levels of ω6 LC-PUFA and intermediate fatty acids beyond ALA. Earlier studies indicated that co-production of EPA and DHA in seed tended to result in reduced accumulation of at least one of these fatty acids. We decided to focus on the production of the maximal level of DHA rather than co-producing EPA and DHA for a number of reasons. Commodity fish oil (e.g., anchovy) contains a higher level of EPA compared to DHA (16–18% vs 12%) so from a purely source-oriented perspective DHA is the rarer molecule. DHA also appears to be more difficult for aquaculture species to synthesize *de novo* and must be exogenously supplied during feeding, whereas EPA can be synthesized from ALA at a higher rate (Emery et al., 2016; Bou et al., 2017). Finally, the linear nature of DHA biosynthesis in seed means that a successful DHA production pathway can easily be converted to an EPA or DPA production pathway by omitting the Δ5-elongase or Δ4-desaturase, respectively. In fact, we have initiated development of an EPA-only pathway in canola which will be the subject of a separate study.

Key considerations in achieving a high level of DHA accumulation included (1) avoiding excessive ‘pool shuffling’ between acyl-CoA and acyl-PC pools, (2) use of a Δ6-desaturase with ω3 preference, (3) promoter timing designed to reduce accumulation of intermediate fatty acids, and (4) duplicating parts of the pathway to increase gene expression and efficiency. Rapid testing of alternative construct designs in the somatic embryo assay facilitated these goals.

We elected to use the Δ6-desaturase pathway (Figure 1) with supplementation of the native FAD2 (Δ12-desaturase) and FAD3 (Δ15-desaturase) activities to increase the production of the substrate ALA. Accumulation of intermediate fatty acids and higher flux (> 78%) through the post SDA part of the pathway was achieved by reducing the amount of fatty acid ‘pool shuffling’ between acyl-CoA (where elongation occurs) and acyl-PC (where desaturation typically occurs in plant metabolism) (Abbadi et al., 2004; Robert et al., 2005). In choosing the front-end desaturases we deliberately selected desaturases that act on fatty acid substrates in the acyl-CoA pool. For example, the acyl-CoA desaturation ability of the *M. pusilla* Δ6-desaturase was described in Petrie et al. (2010). In addition, Yilmaz et al. (2017) demonstrated that the *P. lutheri* Δ4-desaturase, a gene that is very closely related to the *P. salina* Δ4–desaturase used in this study, was able to desaturate acyl-CoA substrates to reduce pool shuffling. This resulted not only in the desaturated fatty acids but also the subsequent elongated fatty acids being produced with high efficiency, thus avoiding intermediate fatty acid accumulation. Based on a phylogenetic analysis of amino acid sequences (data not shown), the *P. salina* Δ5-desaturase clusters with other demonstrated acyl-CoA desaturases and is therefore predicted to use ETA-CoA as its primary substrate. In this study, the retention of fatty acid intermediates in the acyl-CoA pool provided for very efficient Δ6- and Δ5-elongase steps. Furthermore, we achieved an extremely high long-chain ω3 to ω6 ratio by limiting the amount of ω6 pathway flux with a Δ6-desaturase that had a strong preference for the ω3 substrate, ALA (Petrie et al., 2010). We also used an ω3-desaturase that converted LA to ALA (Zhang et al., 2008).

A common feature of the fatty acid profiles described in Figure 4 and Table 1 is that Δ6-desaturation is the rate-limiting step for this engineered pathway with only about half the ALA converted to SDA. There are several possible reasons for this. First, it could simply be a matter of low transcript abundance leading to low protein levels. This is unlikely, because although there is a clear gene dosage effect, high copy number events, possibly resulting in higher transcript levels, somewhat improved but did not overcome rate limitation at this step. It is also noteworthy that the majority of evidence suggests that fatty acid desaturation activity is regulated primarily at the post-translational level and hence higher transcript levels might not necessarily result in higher desaturase activity or protein levels (O’Quin et al., 2010). Interestingly, in a separate but related study, the *M. pusilla* Δ6-desaturase protein was the least abundant of the three algal front-end desaturases used in our DHA pathway. For example, *P. salina*-Δ4D protein levels ranged from up to 1,500 ng/mg total seed protein in mature seed and up to 5,600 ng/mg seed protein in developing transgenic canola seed. In contrast, *M. pusilla* Δ6-desaturase protein levels ranged from 220 ng/mg total seed protein in mature seed and up to 870 ng/mg total seed protein in developing transgenic canola seed (Colgrave et al., 2019b). Another possibility is that the *M. pusilla* Δ6-desaturase has low activity from a functional perspective. In fact, a study published recently indicates that there is a constraint imposed by increased preference for ALA over LA by *M. pusilla* Δ6-desaturase which results in the enzyme inherently displaying lower activity (Li et al., 2020). Finally, it is possible that the *M. pusilla* Δ6-desaturase enzyme has a very strong preference for acyl-CoA substrates, such that the bulk of the naturally produced acyl-PC ALA is unavailable for conversion until it enters the acyl-CoA pool. It is also conceivable that much of the acyl-PC ALA simply ends up in triacylglycerol via the action of PDAT without ever entering the acyl-CoA pool (Pan et al., 2013). Further work is required to develop strategies to increase activity at this rate-limiting step.

This study further highlighted the importance of construct design and promoter selection when introducing new metabolic pathways to plant hosts (Cheng et al., 2010). For example, synthesis of intermediate fatty acids in the absence of an enzyme that can perform further conversion might result in the removal of the intermediate fatty acid from the metabolically active lipid pool into storage lipids where they are no longer accessible as a substrate. We used relatively late promoters (seed storage protein promoters Cnl2 and napin) to express the first committed step of synthesis, Δ6-desaturation. An example of this principle can be seen in the relatively poor Δ6-elongation observed in GA7_mod-F embryos (Supplementary Table 2): although the Δ6-desaturation was higher following the addition of a FAE1::Δ6-desaturase expression cassette, the subsequent conversion of SDA to ETA was lower, indicating that the additional SDA was not entirely available for conversion.

To speed up the process of selecting a construct for large-scale transformation, we used a rapid somatic embryo assay instead of testing each construct variant in canola seed. Constitutive expression of the LEC2 transcription factor during the early stages of tissue culture can result in the production of somatic embryos with seed-like fatty acid profiles (Stone et al., 2008). This system provides the activation of seed-specific promoters, allowing rapid assessment of seed-specific construct function after co-transformation (Petrie et al., 2013). The fatty acid profiles achieved in the somatic embryos described in this study shared some characteristics despite the varying DHA levels: a very high ω3 to ω6 LC-PUFA ratio, high total ω3 PUFA levels, high conversion efficiency from SDA through to DHA, and low accumulation of intermediate fatty acids. These profiles indicate that the conversion efficiency from SDA through to DHA is remarkably high, with little loss of substrate along the pathway. This is likely due to the selection of LC-PUFA desaturases which are capable of acting in the acyl-CoA pool, resulting in both elongation and desaturation in the same metabolite pool as described above.

Interestingly, expression of the GA7_mod-B construct was found to be highly stable when integrated into the canola genome, even with the twin T-DNA insertions in an inverted repeat arrangement on the A05 chromosome. DHA levels have been remarkably stable in both glasshouse and field trials over several years and up to the T_7_ generation, under a wide range of growing conditions (Table 2). The effect of the A02 partial construct locus is also interesting: this fragment is effectively acting as a ‘booster’ for the full pathway insertion in chromosome A05 which increased gene dosage without increasing the number of genes in the construct. Notably, DHA levels increased from 2.1% in the absence of the A02 partial construct to 9.7% when present with the A05 locus (Table 1). The use of partial pathway insertions to provide multiple copies of some genes should not be overlooked when engineering complex pathways, especially for the specific use of increasing flux through bottlenecks. In addition, proteomics combined with a targeted LC-MS/MS approaches were applied to simultaneously measure the relative low abundance of seven transgenic enzymes involved in the docosahexaenoic acid (DHA) biosynthetic in DHA canola from multiple filed trials, which are all membrane-bound (Colgrave et al., 2019b). The results demonstrated that the seed-specifically expressed enzymes that drive the production of DHA were only detected in mature and developing seed of DHA canola. *In vitro* protein digestibility analysis using a strategy with two-stage digestion including simulated gastric fluid trypsin showed that these proteins were rapidly degraded by > 95% within 5 min (Colgrave et al., 2019a).

The field trial results confirm that NS-B50027-4 retains the seed yield of the parental line, with only a slight reduction in total seed oil content. However, the value proposition for this crop is very different from conventional canola, and the value of the oil is expected to compensate for the oil content reduction. Previous research has shown either no significant effect of LC-PUFA expression (14% total LC-PUFA) on seed oil content (Napier et al., 2015), or an oil content reduction of 3.0–8.3% in plants expressing 16–17% EPA (Usher et al., 2017). Further research to incorporate the trait in other germplasm, high-yielding open-pollinated and hybrid lines, may be undertaken. Although NS-B50027-4 is primarily adapted to Australian conditions, the field results indicated that it performed adequately in Canada and the United States, with reasonable grain yield and, more importantly, consistent LC-PUFA yields.

The oil from NS-B50027-4 has several unique features: it is relatively high in DHA, a highly beneficial LC-PUFA for dietary supplementation (Napier et al., 2019), but also contains a small amount of EPA and a significant amount of DPA, a fatty acid in which there appears to be increasing interest from the medical community (Kaur et al., 2016). In addition, the oil contains a high level of ALA (ca. 20%, compared to 10% in typical canola oil). This provides a substantially increased ω3:ω6 ratio, with concomitant health benefits. In addition, the predominance of DHA at *sn*-1 and *sn-*3 on TAG suggests it will be highly available upon ingestion. The long-term stability of the LC-PUFA in NS-B50037-4 suggests there will be little loss of the key fatty acids during storage and transportation of grain from this crop, removing some of the logistical concerns with protecting LC-PUFA from premature oxidation.

Omega-3 canola oil is expected to have multiple applications in human and animal nutrition. The high DHA and total ω3 levels achieved suggest that this oil is particularly suitable as an ingredient in aquaculture feed. Vegetable and poultry oils with a low ω3 to ω6 ratio have become a major energy source in commercial aquaculture feeds, resulting in a significant reduction in the ω3 to ω6 ratio in farmed Atlantic salmon compared to wild-caught fish (Strobel et al., 2012) or fish raised on high fish-oil diets (Nichols et al., 2014). Dietary supplementation with this ω3 canola oil results in a high ω3 and DHA content in fillets of Atlantic salmon (Ruyter et al., 2019), with consequent benefits to consumers of such fillets. This and a more recent study (Betancor et al., 2018) indicated no negative effects on fish health, growth and survival, confirming that such oils are safe and effective replacements for fish oils in aquaculture diets.

The optimal levels of EPA and DHA for fish growth and health are not yet clearly established, although the low level of EPA in the omega-3 canola oil does not appear to be a detriment to fish nutrition. Indeed, there is evidence that EPA may not be required by Atlantic salmon under certain conditions (Bou et al., 2017; Usher et al., 2017). Evidence for forward conversion of ALA to EPA and DHA has been demonstrated in Atlantic salmon (Stubhaug et al., 2005; Sanden et al., 2011; Bou et al., 2017) and rainbow trout (Turchini et al., 2011; Hixson et al., 2014), particularly when the LC-PUFA are incorporated in the feed at low levels, as occurs with the presence and use of any fish meal in the feed. The high ALA content of the omega-3 canola oil may therefore alleviate its relatively low EPA content, making it a suitable replacement for fish oil in aquaculture diets. Conversion of EPA to DHA is very inefficient in humans (Kitessa et al., 2014), with use of a DHA-containing oil being seen therefore as considerably more beneficial from both an animal (farmed fish) and then the resulting human nutrition perspective.

Oil from line NS-B50027-4 may also provide a direct increase in LC-PUFA availability for human nutrition and health. Based on a Recommended Daily Intake (RDI) of 500 mg (Nichols et al., 2010), as proposed by various national health bodies, a global population reaching 8 billion by 2025, and assuming that fish contain on average 0.3 g (range 0.2–3.5 g) of EPA and DHA per 100 g of edible flesh, it is estimated that the current global fish harvest (93 million tons per annum) will fall well short of meeting this requirement (Naylor et al., 2009). In addition to the nutritional benefits occurring for both the farmed fish and the human consumers, the use of omega-3 canola oil in aquaculture feeds (Ruyter et al., 2019) will also provide substantial environmental benefits through the reduced pressure on the wild harvest fisheries which presently no longer meet the market demand.

Cultivation of the DHA canola crop and human and animal consumption of the oil in Australia and cultivation in USA has been approved by the relevant regulators (FSANZ, 2017; OGTR, 2018; USDA, 2018). Also, the successful engineering of high levels of DHA in canola opens the door to large-scale production of other important LC-PUFA such as EPA and DPA. These fatty acids are intermediates in the metabolic pathway we have used to produce DHA in this study. For example, the use of different versions of the GA7 construct may yield fish oil-like levels of EPA and even higher levels of the relatively rare fatty acid DPA, or possibly further increase the amount of DHA that can be produced in canola seed (Shrestha et al., 2018).

## Data Availability Statement

The sequences of the transgene insert and *B. napus* flanking regions at A02 and A05 were deposited in GenBank under accession numbers MP172039 and MP172040.

## Author Contributions

JP, X-RZ, PN, RF, MD, and SS made substantial contributions to the conception and design of the work, analysis and interpretation of data, and extensive drafting or editing of the manuscript. JM, PS, YK, WG, GL, MM, RM, QL, LT, CS, and NC analyzed and interpreted data. AL, SB, GB, and NG made substantial contributions to the design of the work and analyzed and interpreted data. AG made substantial contributions to the conception of the work.

## Conflict of Interest

The authors declare that this study received funding partially from CSIRO, Nuseed and GRDC. CSIRO is not an affiliate of any of the Nuseed entities. The Nuseed entities had the following involvement with the study: Authors AL, GB and NG participated in event selection and were responsible for all breeding, line advancement and genetic analysis; JM and CS were responsible for oil and fatty acid analysis; WG was responsible for molecular analysis and marker development; MD was responsible for overall project planning and writing parts of the manuscript. The remaining authors declare that the research was conducted in the absence of any commercial or financial relationships that could be construed as a potential conflict of interest.

## Footnotes

1 http://www.bioinformatics.nl/cgi-bin/primer3plus/primer3plus.cgi

2 http://www.cstl.nist.gov/strbase/AutoDimerHomepage/AutoDimerProgramHomepage.htm

3 http://bio-bwa.sourceforge.net/

4 http://samtools.sourceforge.net/

5 https://ics.hutton.ac.uk/tablet/

6 http://www.genoscope.cns.fr/blat-server/cgi-bin/colza/webBlat

7 http://brassicadb.org/brad/blastPage.php

8 http://plants.ensembl.org/Brassica_oleracea/Tools/Blast?db=core

9 http://doua.prabi.fr/software/cap3

10 http://primer3plus.com/cgi-bin/dev/primer3plus.cgi

11 http://www.nvtonline.com.au
