# Supplementary material for: Development of a Brassica napus (Canola) Crop Containing Fish Oil-Like Levels of DHA in the Seed Oil
Source: Front Plant Sci. 2020 Jun 12;11:727. doi: 10.3389/fpls.2020.00727 (PMC7303301; doi:10.3389/fpls.2020.00727)
Supplement: Supplementary file 1 [file Data_Sheet_1.docx]

**Supplementary Table 1** | PCR primers designed to span the supplied T-DNA sequence. Primers were synthesised with the Illumina adaptor PE1 sequence added to the tail of each primer.

| DEPI_Petri_001_F1: TCGCGTTGGCAGCATCACCC | DEPI_Petri_001_F44: AGGCTACTGGACACATGACC |
| --- | --- |
| DEPI_Petri_001_F3: CACTCCATCGACATATCGG | DEPI_Petri_001_F45: TACAACGCTAGACCTGCTTC |
| DEPI_Petri_001_F5: AAGTGTTTTGGCTCTCAGG | DEPI_Petri_001_F46: CAATTGAGGAGCGAGTGCAC |
| DEPI_Petri_001_F7: ACAAGGACGTGAAGATCACC | DEPI_Petri_001_F47: CTCGTGTGGGTTCTATCTCG |
| DEPI_Petri_001_F9: CAAGTCAGAGGTGGCGAAAC | DEPI_Petri_001_F48: CCATGGAATTTGCTCAACC |
| DEPI_Petri_001_F10: TTGGTATCTGCGCTCTGCTG | DEPI_Petri_001_F49: GATCACTTATGCTGCTCCAGG |
| DEPI_Petri_001_F11: GCGGGAAACGACAATCTGC | DEPI_Petri_001_F50: TACTACATGAAGCACCACGC |
| DEPI_Petri_001_F12: TCAGATCTTCCAAGGCCTCG | DEPI_Petri_001_F51: GTGGAAAAGGAAGGTGGCTC |
| DEPI_Petri_001_F13: TCTCAGCCTTTCCGTTAACG | DEPI_Petri_001_F52: ATCGCTTACGCTGGACCTTGG |
| DEPI_Petri_001_F14: AAACGAGCATCCAAGAAGCC | DEPI_Petri_001_F53: TCCTAGACCTGTTAGACCAG |
| DEPI_Petri_001_F21: GTCAGAGGGTATGCCAATCAG | DEPI_Petri_001_F54: CACCACAATATATCCTGCCAC |
| DEPI_Petri_001_F22: TGCTGGAACTCTTGGATACG | DEPI_Petri_001_F55: TCACCACTCGATACAGGCAG |
| DEPI_Petri_001_F23: CACACTCTTCTCATCTGGTC | DEPI_Petri_001_F56: CAGCTCGGCACAAAATCACC |
| DEPI_Petri_001_F24: GACTCTCACTACTCGATCGC | DEPI_Petri_001_F57: TCGTCAGCCATTAATCGTCC |
| DEPI_Petri_001_F25: CAATTACCATACCATGCCTCC | DEPI_Petri_001_F58: ACCATCCTGCTCCATACTCGC |
| DEPI_Petri_001_F26: TCAGGGAAGATGTGGATGGC | DEPI_Petri_001_F60: AAGGACGGCAAGACTGTTGG |
| DEPI_Petri_001_F27: GCCTAACGAGCATGCTACTTG | DEPI_Petri_001_F62: AATTGCTCTTGTCGATCGAC |
| DEPI_Petri_001_F28: CTATGTTACTAGATCGGTCG | DEPI_Petri_001_F64: AATGCGGATAGCGTGAGATAC |
| DEPI_Petri_001_F29: TATAAACCGAACCAACTCGG | DEPI_Petri_001_F66: CCAATATAGGCGAAGTCACC |
| DEPI_Petri_001_F30: CATAACATTCCATCAGCCTC | DEPI_Petri_001_R1: CAATTATGGGTGATGCTGCC |
| DEPI_Petri_001_F31: AGAACCACACGAGCATGTGC | DEPI_Petri_001_R2: TCATGTCCTTTTCCCGTTCC |
| DEPI_Petri_001_F32: ATCGATAGTAGCAGCAGCAC | DEPI_Petri_001_R3: AATCATACAGCTCGCGCGG |
| DEPI_Petri_001_F33: GAAAGCAGAATGTCCACACTC | DEPI_Petri_001_R4: ATCGTTGCGCCACATCTAGG |
| DEPI_Petri_001_F34: ATGGGCTACACGTGGGTTC | DEPI_Petri_001_R5: GGTTTATCCGTTCGTCCAT |
| DEPI_Petri_001_F35: TCAGTTGGGATAGCAGATCC | DEPI_Petri_001_R6: TCCACTAGCAGATTGTCGTT |
| DEPI_Petri_001_F36: ACCCGAAGCCTCATGAAAGC | DEPI_Petri_001_R7: GAAGATCTGAGTCGAACGGG |
| DEPI_Petri_001_F37: ATCCCGTCATTTGCATCCAC | DEPI_Petri_001_R8: TGCTTCTACTGCTCCTGTTG |
| DEPI_Petri_001_F38: CTTGAGAGACACCTGCACTTC | DEPI_Petri_001_R9: GGTGTTTCAGAGTCTTCGTG |
| DEPI_Petri_001_F39: GGAATACCATAGAAGGGCTTG | DEPI_Petri_001_R10: CGGTAGAAGTTGGCGAAGAG |
| DEPI_Petri_001_F40: GGTGAGACTATGTACGCTTTC | DEPI_Petri_001_R11: ACATCGTAGTGAAGTCCGTG |
| DEPI_Petri_001_F41: GACATATGTACGCTCTTGG | DEPI_Petri_001_R12: AGAGCCAGCACTTCGATAGC |
| DEPI_Petri_001_F42: ACGAGGCCTTGGAAGATCTG | DEPI_Petri_001_R13: CACGTGTAGCCCATGCAAAG |

**Supplementary Table 2** | Representative fatty acid conversion efficiencies in *Brassica napus* somatic embryos generated by co-transformation of a 35S::LEC2 vector with a seed-specific GA7 construct variant. Conversion efficiencies are calculated as *sum(products)/sum(substrate + products)*. DHA is given as a percentage of the somatic embryo total fatty acids.

|  | **Δ12-D %** | **Δ15-D %** | **Δ6-D %** | **Δ6-E %** | **Δ5-D %** | **Δ5-E %** | **Δ4-D %** | **DHA** |
| --- | --- | --- | --- | --- | --- | --- | --- | --- |
| **GA7_mod-B** | 89.9 | 78.0 | 24.1 | 91.6 | 95.5 | 96.5 | 81.7 | 8.7% |
| **GA7_mod-D** | 98.3 | 85.2 | 26.3 | 69.8 | 93.1 | 97.3 | 68.1 | 6.4% |
| **GA7_mod-E** | 88.2 | 77.9 | 24.2 | 86.0 | 92.3 | 91.0 | 70.5 | 6.1% |
| **GA7_mod-F** | 90.4 | 81.3 | 32.9 | 65.7 | 98.2 | 84.0 | 86.7 | 8.3% |
| **GA7_mod-G** | 63.8 | 59.7 | 19.6 | 83.1 | 92.7 | 97.1 | 88.7 | 3.6% |

Note: The results are based on pooled 4-5 similar size and stage of somatic embryos and only to show the trends.

**Supplementary Table 3** | Grain yield (T.ha^-1^) and oil content (percentage of seed weight) of NS-B50027-4 (T_3_ and T_5_ generations), the A02 and A05 segregant lines, and comparator varieties. Sites were located in Victoria, Australia (Ararat, Toolondo, Douglas, Kaniva, and Gymbowen), Saskatchewan, Canada (Vanguard), and Alberta, Canada (Coalhurst). A05 and A02 locus entries are NS-B50027-4 F2-derived F6 lines. ATR Bonito, ATR Stingray, AV Garnet and Monola 515TT are commercial Australian varieties; DK7444 (Roundup Ready) and LL130 (InVigor) are high-yielding Canadian hybrids. P level = <0.101 for all entries except Coalhurst where P level = <0.119. Each row shows individual data points from 3-5 biological repeats of bulk seeds at multiple field trial sites in two years.

|  | **2015** | | | | | | **2016** | | | | | | | | | |
| --- | --- | --- | --- | --- | --- | --- | --- | --- | --- | --- | --- | --- | --- | --- | --- | --- |
|  | **Ararat** | | **Toolondo** | | **Douglas** | | **Kaniva** | | **Gymbowen** | | **Ararat** | | **Vanguard** | | **Coalhurst** | |
|  | **Yield** | **Oil** | **Yield** | **Oil** | **Yield** | **Oil** | **Yield** | **Oil** | **Yield** | **Oil** | **Yield** | **Oil** | **Yield** | **Oil** | **Yield** | **Oil** |
| NS-B50027-4 T3 | 1.18 | 35.1 | 1.17 | 38.2 | 1.64 | 37.3 | 2.94 | 42.5 | 3.26 | 42.5 | 2.54 | 44.9 | 1.01 | 43.0 | 3.26 | 37.4 |
| NS-B50027-4 T5 |  |  |  |  |  |  | 2.99 | 41.7 | 3.22 | 41.0 | 2.35 | 42.9 | 0.97 | 42.8 | 3.58 | 38.2 |
| A05 Locus |  |  |  |  |  |  | 3.63 | 41.2 | 3.44 | 42.2 | 2.27 | 44.7 |  |  |  |  |
| A02 Locus |  |  |  |  |  |  | 4.37 | 47.2 | 4.49 | 46.2 | 3.65 | 48.8 |  |  |  |  |
| AV Jade | 1.01 | 39.4 | 0.8 | 42.2 | 1.22 | 42.5 | 4.11 | 47.0 | 3.77 | 46.4 | 3.25 | 50.4 | 1.25 | 48.0 | 3.05 | 41.4 |
| ATR Bonito | 1.33 | 39.6 | 1.28 | 43.4 | 1.74 | 42.6 | 3.64 | 46.8 | 3.50 | 46.1 | 3.11 | 49.0 | 1.09 | 48.6 | 2.80 | 41.8 |
| ATR Stingray | 1.5 | 39.9 | 1.44 | 42.8 | 1.67 | 41.1 | 3.90 | 45.6 | 3.73 | 46.1 | 2.87 | 48.5 | 0.67 | 45.3 | 2.75 | 41.1 |
| AV Garnet | 1.18 | 36.4 | 1.63 | 41.8 | 2.37 | 41.5 | 4.26 | 45.7 | 4.31 | 45.2 | 3.60 | 48.0 | 1.42 | 47.9 | 3.71 | 40.5 |
| Monola 515TT | 1.23 | 39.3 | 1.09 | 42.1 | 1.55 | 42.2 | 2.83 | 45.8 | 3.66 | 44.3 | 3.07 | 47.2 | 0.80 | 46.6 | 2.40 | 39.5 |
| DK7444 |  |  |  |  |  |  |  |  |  |  |  |  | 1.74 | 51.8 | 3.83 | 45.3 |
| LL130 |  |  |  |  |  |  |  |  |  |  |  |  | 1.63 | 49.4 | 4.22 | 42.6 |
|  | | | | | | | | | | | | | | | | |
| LSD^1^ | 0.15 | 0.62 | 0.4 | 0.7 | 0.2 | 0.73 | 0.28 | 1.07 | 0.37 | 0.65 | 0.38 | 0.69 | 0.18 | 0.62 | 0.95 | 1.18 |
| CV%^1,2^ | 7.1 | 0.8 | 16 | 0.9 | 6.4 | 0.9 | 3.9 | 1.2 | 4.9 | 0.7 | 6.3 | 0.7 | 8.15 | 0.7 | 15.61 | 1.4 |

^1^Least Significant Difference (LSD) and Coefficient of Variation (CV) are used for multiple location/year data analysis with GenStat (Version 17).

**^1^**CVs were low (1.5% or less) for oil content, and in almost all cases under 10% for yield.
