# Supplementary material for: Development of a Brassica napus (Canola) Crop Containing Fish Oil-Like Levels of DHA in the Seed Oil
Source: Front Plant Sci. 2020 Jun 12;11:727. doi: 10.3389/fpls.2020.00727 (PMC7303301; doi:10.3389/fpls.2020.00727)
Supplement: Supplementary file 2 [file Data_Sheet_2.pdf]

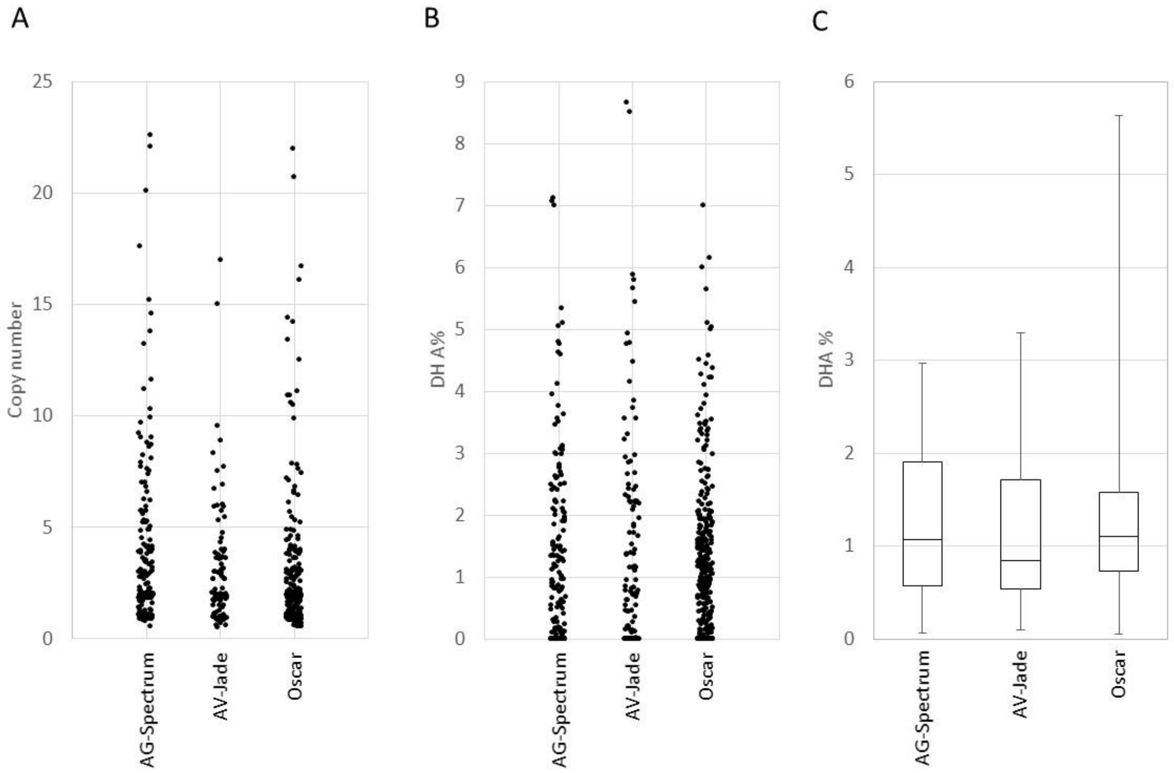

SUPPLEMENTARY FIGURE 1 | Characterisation of T0 transgenic lines. (A) Copy of T-DNA insertion in 193, 108 and 449 transgenic lines from varieties AG-Spectrum, AV-Jade and Oscar. (B) T1 seed DHA levels in these T0 lines (each dot shows the highest DHA level from 5 single seeds per line). (C) Box plot of T1 seed DHA levels in those T0 lines with 1-2 copies of T-DNA insertion, n=22, 21 or 119 for AG-Spectrum, AV-Jade or Oscar.

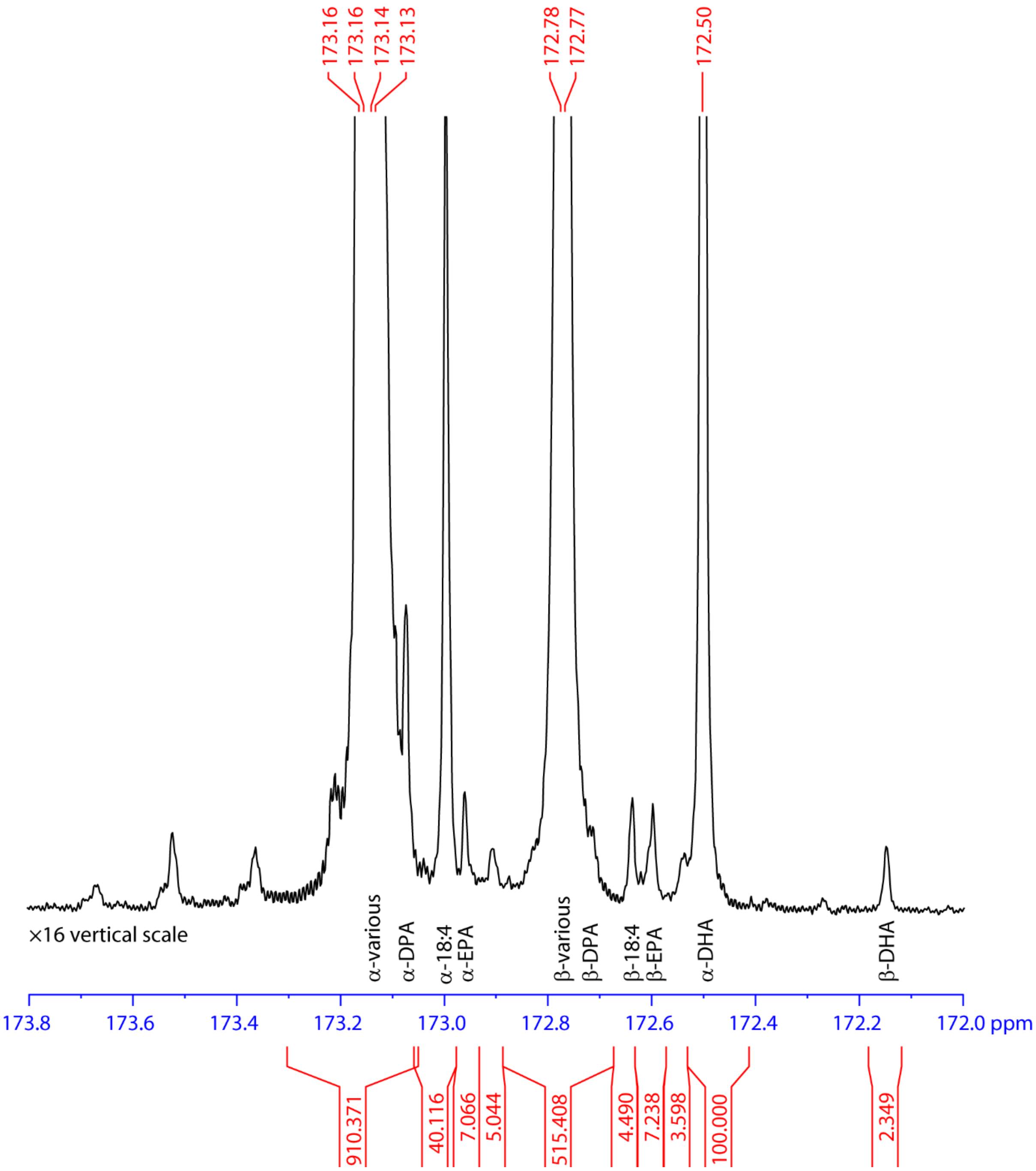

SUPPLEMENTARY FIGURE 2 | NMR scan showing positional distribution of DHA and other PUFA and LC-PUFA in GA7_mod-B transgenic *Brassica napus* seed oil. 97% of the DHA was present at the *sn*-1,3 positions (α-DHA) of the triacylglycerol.

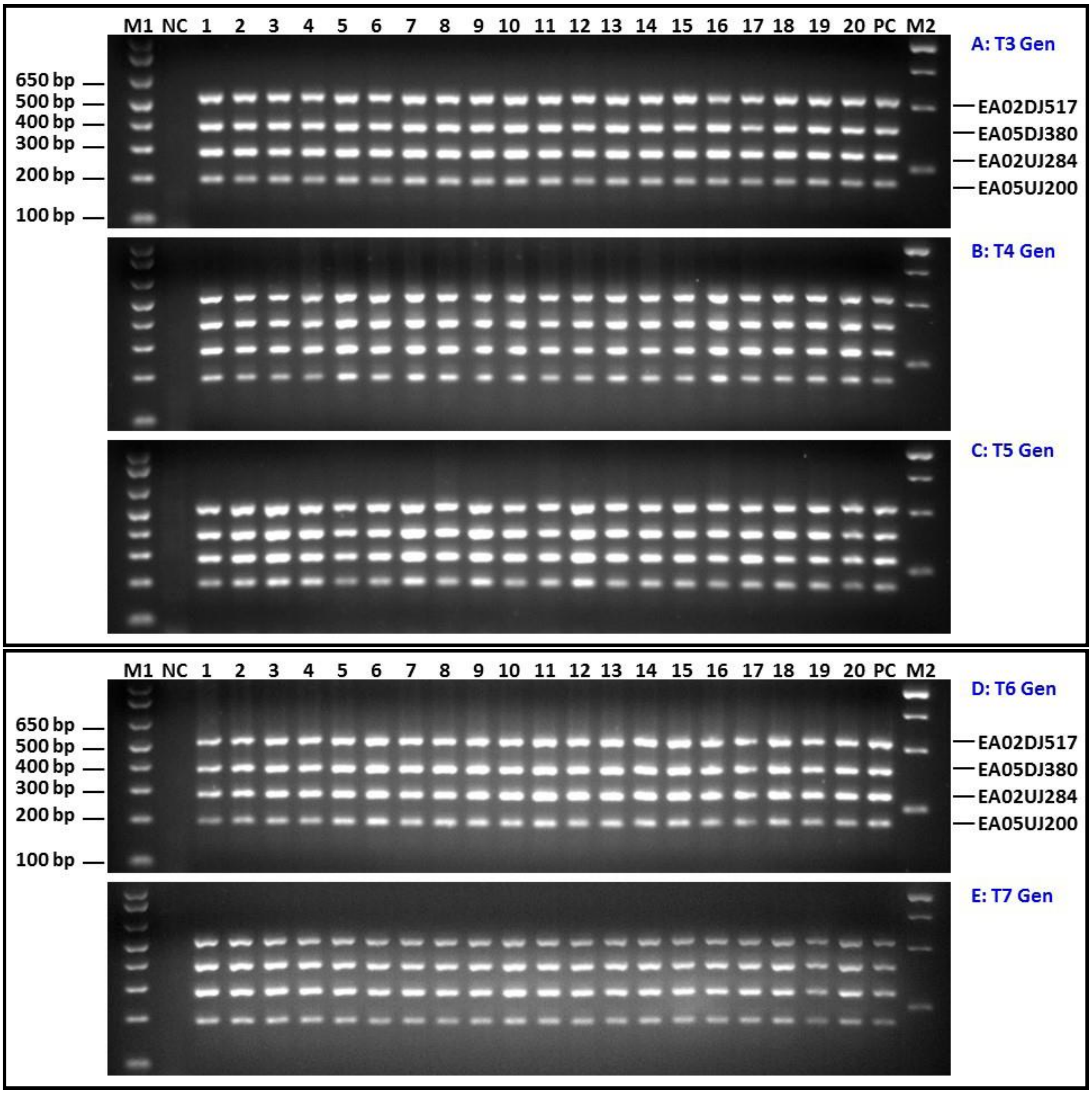

SUPPLEMENTARY FIGURE 3 | PCR of all four borders of five generations of DHA canola. PCR amplicon sizes of DHA canola T_3 (**A**), T_4 (**B**), T_5 (**C**), T_6 (**D**) and T_7 (**E**) seeds genotyped with four assays (EAD02DJ517, EA05DJ380, EA02UJ284 and EA05UJ200). Each assay targeted one junction region of the two T-DNA inserts. All 100 seeds from T_3 to T_7 generations (20 seeds / generation) had the same expected amplicon sizes of 517 bp, 380 bp, 284 bp and 200 bp for EAD02DJ517, EA05DJ380 EA02UJ284 and EA05UJ200, respectively. M1: 1 Kb+ DNA Ladder (Invitrogen): M2: GeneRuler 1 Kb DNA Ladder (Thermo Scientific); NC: negative (non-GMO) control AV Jade; PC: DHA canola positive control (T_5 seed B0005-027-18-20-12-19). Numbers 1 to 20 indicated 20 DHA canola seeds from each of T_3 to T_7 generations.
